# Outcomes of Deceased Donor Kidney Transplantation Using Expanded Criteria Donor Kidneys Following Pulsatile Preservation

**DOI:** 10.7759/cureus.5091

**Published:** 2019-07-07

**Authors:** Amit Basu, Lisa M Rosen, Henkie P Tan, Joanna Fishbein, Christine M Wu, Joseph B Donaldson, Susan Stuart, Nirav A Shah, Jerry McCauley, Abhinav Humar, Ron Shapiro

**Affiliations:** 1 Surgery, Jamaica Hospital Medical Center, New York, USA; 2 Statistics, Hofstra University, Hempstead, USA; 3 Surgery, Thomas E Starzl Transplantation Institute, University of Pittsburgh Medical Center, Pittsburgh, USA; 4 Biostatistics, Feinstein Institute of Medical Research, Manhasset, USA; 5 Nephrology, Thomas E Starzl Transplantation Institute, University of Pittsburgh Medical Center, Pittsburgh, USA; 6 Bio-Informatics, Thomas E Starzl Transplantation Institute, University of Pittsburgh Medical Center, Pittsburgh, USA; 7 Miscellaneous, Organ Procurement Organization, Center for Organ Recovery and Education, Pittsburgh, USA; 8 Medicine, Sidney Kimmel Medical College at Thomas Jefferson University, Philadelphia, USA; 9 Surgery, Recanati-Miller Transplantation Institute, the Mount Sinai Hospital, New York, USA

**Keywords:** deceased donor renal transplantation, machine preservation, expanded criteria donor, cold ischemia time, cold storage, organ procurement organization, kdpi: kidney donor profile index, delayed graft function, patient survival, graft survival

## Abstract

Aim

We compared the outcomes of transplanting expanded criteria donor (ECD) kidneys undergoing machine perfusion (MP) versus cold storage (CS).

Material and methods

Data on all expanded criteria deceased donor kidney transplants performed at the University of Pittsburgh Medical Center from January 2003 through December 2012 were collected from an in-house electronic repository. There were 78 patients in the MP group and 101 patients in the CS group. The majority of the ECD kidneys were imported from other organ procurement organizations: 69 of 73 in the MP group (94.5%, 5 from unknown sources); and 90 of 99 in the CS group (91%), 2 from an unknown source). Most of the patients in the MP group (77 of 78) received a combination of MP and static CS. MP was performed just prior to transplantation in all MP patients. We used descriptive statistics to characterize our sample. We used logistic regression analysis to model the binary outcome of delayed graft function (DGF; i.e., “yes/no”) and Cox (proportional hazard) regression to model time until graft failure. The Kaplan-Meier product-limit method was used to estimate survival curves for graft and patient survival.

Results

A total of 179 transplants were done from ECD donors (MP, 78; CS, 101). The mean static cold storage time was 14 ± 4.1 hours and the mean machine perfusion time was 11.2 ± 6.3 hours in the MP group. The donor creatinine was higher (1.3 ± 0.6 mg/dl vs. 1.2 ± 0.4 mg/dl, p = 0.01) and the cold ischemia time was longer (28.9 ± 10 hours vs. 24 ± 7.9 hours, p = 0.0003) in the MP patients. There were no differences between the two groups in DGF rate (20.8% [MP] vs. 25.8% [CS], p = 0.46), six-year patient survival (74% [MP] vs. 63.2% [CS], p = 0.11), graft survival (64.3% [MP] vs. 51.5% [CS], p = 0.22), and serum creatinine levels (1.5 mg/dl vs. 1.5 mg/dl) on univariate analysis. On unadjusted analysis, MP subjects without DGF had longer graft survival compared to CS subjects with DGF (p < 0.0032) and MP subjects with DGF (p < 0.0005). MP subjects without DGF had longer death-censored graft survival compared to CS subjects with DGF (p < 0.0077) and MP subjects with DGF (p < 0.0016). However, on regression analysis, MP subjects had longer graft survival than CS subjects when DGF was not present. MP subjects without DGF had longer patient survival compared to CS subjects with DGF (p < 0.0289), on unadjusted analysis. MP subjects had a reduced risk of graft failure (hazard ratio [HR], 0.34; 95% confidence interval [CI], 0.17, 0.68) and death-censored graft failure (HR, 0.44; 95% CI, 0.19, 1.00), compared to CS subjects when DGF was not present.

Conclusions

Reduction of DGF rates for imported ECD kidneys is vital to optimize outcomes and increase their utilization. One strategy to decrease DGF rates may be to reduce static CS time during transportation, by utilizing a portable kidney perfusion machine.

## Introduction

The expanded criteria donor (ECD) policy in renal transplantation was implemented in October 2002 [1]. The ECD kidney has a relative risk of graft failure > 1.7 times that of a reference group of ideal donor kidneys (i.e., the standard criteria kidneys [SCD]). Using this definition, based on the increased risk of graft loss, all donors over 60 years of age and donors aged 50 to 59 years with at least two of the following three medical criteria were identified as ECD: 1) cerebrovascular accident as the cause of death; 2) history of hypertension; 3) terminal creatinine levels > 1.5 mg/dL [1]. Although the expected benefits associated with deceased donor kidneys vary widely based on characteristics of the organ transplanted, even higher risk kidneys are associated with a significant survival advantage compared to maintenance dialysis for transplant candidates [2].

There continues to be a significant imbalance between the supply of kidneys for transplantation versus the demand. In a recent Scientific Registry of Transplant Recipients (SRTR) report, the national waiting list for kidney-alone recipients was 95,456 by the end of 2016 [3]. A total of 18,836 kidney transplants were performed in 2016, 13,501 of which used kidneys from deceased donors (DDs). More than 4,800 patients died on the waiting list in 2016, and another 4,411 were removed from the list due to their deteriorating medical condition. Hence, efforts must be directed at optimizing and maximizing the utilization of DD kidneys, including the kidneys procured from ECD.

Kidneys from ECD have higher rates of primary non-function, delayed graft function (DGF), and acute rejection [1, 4]. Because of excess ECD recipient mortality in the perioperative period, survival is not equivalent to that of waitlisted patients until 3.5 years post-transplantation. Beyond 3.5 years, survival favors the ECD recipient [5]. Strategies aimed at maximizing the success of transplantation with ECD kidneys are very important. The selective and increased use of kidneys transplanted from ECD is likely to occur if the kidney functions well before recovery, if the morphologic features of the kidney are adequate according to biopsy results, and if the flow to the kidney by pulsatile perfusion is acceptable [6].

Pulsatile perfusion of kidneys helps in three important ways: (1) the selection of kidneys for transplantation as data regarding flow and resistance are important determinants of successful outcomes; (2) hydrostatic effects of machine perfusion (MP) may reduce intrarenal vasoconstriction; (3) additives to MP solutions may ameliorate ischemia-reperfusion injury [7]. There was a lower rate of discard of pumped kidneys in a study done on the SRTR database from October 1999 to June 2005 [7]. A study performed on ECD kidneys reported to the United Network for Organ Sharing (UNOS) database from January 2000 to December 2003 showed a reduced incidence of DGF in kidneys transplanted after MP than after cold storage (CS) [8]. ECD kidneys transplanted after MPs were found to have a lower incidence of DGF than ECD kidneys transplanted after CS [9] in a single center report. By using MP prior to transplantation using ECD kidneys, outcomes similar to SCD kidneys were obtained in the short-term and intermediate-term [10]. MP has also been used in the assessment of ECD kidneys for dual kidney transplantation (DKT) [11].

In an international, randomized controlled trial (RCT), kidneys were randomly assigned from 336 consecutive DDs to MP or CS and were then transplanted. MP significantly reduced the rate of DGF, improved the rate of decline in serum creatinine levels, and improved one-year allograft survival rates [12]. MP preservation reduced the risks of DGF and improved one-year graft survival and function in ECD kidney transplantation as well [13].

To ameliorate the effects of ischemia-reperfusion injury in ECD kidney transplantation, our program started pulsatile perfusion of such DD kidneys in May 2006. In this study, we present the outcomes of transplantation of ECD kidneys preserved using MP compared to the outcomes of transplantation of such kidneys preserved using CS.

## Materials and methods

We conducted a retrospective review of electronic records of all donation-after-brain-death ECD kidney transplantations performed at our center from January 1, 2003, to December 31, 2012. Recipients of other organs besides a kidney transplant, transplants from donation after cardiac death, SCD, living donor transplants, and transplants done for pediatric recipients were excluded. One hundred seventy-nine ECD kidney transplants were recorded and classified according to method of preservation (i.e., CS [N = 101] versus MP [N = 78]). All the kidneys transplanted after MP had variable periods of CS preservation, before or after pumping. These transplants were a combined CS and MP group. All transplant recipients had a minimum of six months of follow-up evaluations.

ECD were classified according to the UNOS definition [1]. The Kidney Donor Profile Index (KDPI) of the transplanted kidneys was calculated retrospectively using the KDPI calculator [14]. DGF was defined as a need for dialysis in the first week after transplantation [8].

The Cockroft-Gault formula was used to estimate donor creatinine clearance (CrCl) to estimate the projected function of the kidney and determine the usage of the kidney as a single or DKT. If the estimated donor CrCl was less than 50 mL/minute or the donor terminal creatinine was greater than 1.9 mg/dl, the kidneys were not transplanted. If the CrCl was between 50 and 70 mL/minute, a DKT was performed placing one kidney on either side or both kidneys on the same side [9].

A pre-reperfusion donor kidney biopsy was used to assist in the evaluation of pre-existing pathology. A donor kidney biopsy showing > 20% glomerulosclerosis or moderate tubular, vascular or interstitial changes, or glomerular thrombi > 10% were considered contraindication to transplantation of the kidney [15-16]. ECD kidneys (either locally procured or imported) were placed on MP, at the transplant surgeon’s request.

MP was performed with a standard dual kidney perfusion system (RM 3 Renal Preservation System, Waters Medical System, Rochester, MN, US) or LifePort kidney transporter (Organ Recovery Systems, Des Plaines, IL, US), which can perfuse one kidney at a time. Kidneys were perfused with Belzer MP Solution (Transmed Corporation, Elk River, MN, US) at 4°C using 40/20 mmHg pressure on the Waters RM3 and 30/15 mmHg on LifePort Kidney Transporter. Additives that were regularly added to the system included mannitol (3 g/L), insulin (40 units per liter), dexamethasone (16 mg/L), ampicillin (1 g/L) and nitroglycerin (4 mg). Additional treatment intervention based on measurements of flow, pressure, and resistance in the circuit included the administration of an extra aliquot or two of nitroglycerin at the surgeon’s request. A flow rate greater than 80 mL/m as well as resistance less than 0.3 (mmHg/mL/min/100g) after a minimum of six hours on MP were considered thresholds for use. Transplantation was performed only after the flow reached 90 mL/minute [9].

No specific upper recipient age limit was considered an absolute contraindication for kidney transplantation; 29 (16.2%) recipients were over age 70 in this series; the oldest was 83 years old.

At the pre-transplant evaluation, all patients underwent medical, psychosocial, and financial evaluation. We did not have a separate ECD kidney transplantation list, because such listing neither mandated nor restricted the recipient to receive an ECD kidney. The decision to accept any kidney whether from an ECD or SCD was made at the time of offer with informed consent. ECD kidneys were transplanted by matching estimated renal functional mass to recipient nephron need and sometimes included the use of DKT (N = 37, 20.7%). In general, recipients with diabetes and/or hypertension were considered for ECD transplantation [5].

Two therapeutic principles were used for immunosuppression: (1) pre-transplant recipient conditioning with the anti-lymphocytic antibody alemtuzumab, and (2) minimal post-transplant immunosuppression with tacrolimus monotherapy [17]. The infusion of alemtuzumab (Campath 1 H) was started after premedication with 1,000 mg of intravenous methylprednisolone to prevent cytokine release syndrome. The patients were started on twice daily tacrolimus (Prograf) on postoperative day one, with a target 12-hour trough level of 10 ng/mL. Renal function was monitored using serum creatinine determination. Oral steroids or secondary agents such as mycophenolate mofetil were added as necessary [17]. Recipients who were hepatitis C antibody positive received preconditioning with daclizumab (Zenapax) or basiliximab (Simulect). Maintenance immunosuppression was with tacrolimus (Prograf) and mycophenolate mofetil (CellCept) in these patients with target tacrolimus trough levels of 12 ng/mL [17].

Surgical site prophylaxis was done using a first-generation cephalosporin prior to incision and two doses postoperatively (unless the patient was allergic to cephalosporins). Antifungal prophylaxis was done using nystatin for three months, and anti-pneumocystis prophylaxis with sulfamethoxazole-trimethoprim (lifelong). Antiviral prophylaxis consisted of valganciclovir for six months, which was extended to one year for transplants from Epstein-Barr virus (EBV)-positive donors to EBV-negative recipients.

All recipients received aspirin (81 mg, once per day). Treatments for hypertension, hyperlipidemia, anemia, and diabetes were initiated or continued as per the usual standard of care. Renal allograft rejection was suspected by an unexplained rise in serum creatinine greater than 0.3 mg/dL or a 25% increase from baseline levels. The diagnosis was confirmed by ultrasonography-guided percutaneous allograft biopsy. Banff Grade 1 rejections were treated with three boluses of intravenous methylprednisolone 500 mg. Grade 1 rejections that persisted or were steroid-resistant were treated with alemtuzumab or Thymoglobulin (RATG). Grade 2 and Grade 3 rejections episodes were also treated with Thymoglobulin (RATG) for five to seven doses.

Cytomegalovirus (CMV) infection was detected by a positive polymerase chain reaction (PCR) assay. The treatment of a CMV infection consisted of a reduction in immunosuppression and administration of oral valganciclovir. CMV PCR was monitored weekly during treatment which was continued for four weeks after the PCR became undetectable.

Detection of the polyomavirus (PV) PCR in urine higher than 10 E +7 or positive plasma PV PCR was used as a trigger for reducing immunosuppression and initiating therapy with weekly intravenous infusions of cidofovir (0.25 mg/kg/dose). Weekly PV PCR testing was continued during treatment. A fall in the PV PCR (urine) to the range 10 E + 6 was used as an indicator to stop cidofovir treatment. Leflunomide (Arava) was added if the PV PCR was unresponsive to cidofovir treatment.

Data were obtained from the in-house transplantation database EDIT (Electronic Data Interface for Transplantation) and from MARS (Medical Archive and Retrieval System). Institutional Review Board (IRB) approval was obtained from the University of Pittsburgh to conduct this study (IRB number PROD 8020405). Informed consent was waived.

Statistical methods

Statistical analysis was done using SAS version 9.5 (SAS Institute, Cary, NC). Descriptive statistics were used to describe our sample (e.g., mean, standard deviation, frequencies, and percentages). 

Logistic regression was used to model the binary outcome of DGF (i.e., “yes/no”), as a function of treatment arm (MP vs. CS), cold ischemia time (CIT), patient age, donor age, first or retransplant, time on dialysis, human leukocyte antigen (HLA) mismatch, enzyme-linked immunosorbent assay (ELISA) anti-HLA class I and ELISA anti-HLA class II. Backward elimination was used to select the final model. Variables considered nonsignificant were removed during backwards selection. The treatment arm was forced into the final model regardless of significance because it was the primary predictor of interest.

The Kaplan-Meier product-limit method was used to estimate survival curves for graft survival and patient survival. The curves were stratified by treatment arm (MP vs. CS) and DGF (yes vs. no). The log-rank test was used to compare survival between groups. Upon finding a significant association, pairwise comparisons with a Tukey-Kramer adjustment were conducted to determine which groups differed from each other.

Cox (proportional hazards) regression was used to model time until graft failure as a function of treatment arm (MP vs. CS), DGF, CIT, patient age, donor age, first or retransplant, time on dialysis, HLA mismatch, ELISA anti-HLA class I and ELISA anti-HLA class II. Backward elimination was used to select the final model. Variables considered nonsignificant were removed during backwards selection. Treatment arm was forced into the final model regardless of significance because it was the primary predictor of interest.

A linear mixed model for repeated measures was used to examine changes in creatinine over time, as well as between treatment arms (MP vs. CS). The outcome of creatinine was log-transformed to meet the standard assumptions necessary for a linear mixed model. Means and their respective 95% confidence intervals were calculated on the log-scale and anti-logged to present the results in their original units. 

## Results

There was a total of 179 subjects, 78 MP (43.6%) and 101 CS (56.4%). Donor and recipient data are presented in Table 1 and Table 2. The donor creatinine was higher and CIT longer in the MP group compared to the CS group.

**Table 1:** Donor and recipient factors **Mann-Whitney, results reported as median (Q1, Q3) ‡‡Evaluated using Wilcoxon Rank Sum Test Abbreviations: MP, machine perfused; CS, cold storage; H/O, history of; CVS, cardiovascular system; CIT, cold ischemia time; KDPI, kidney donor profile index; ELISA, enzyme-linked immunosorbent assay; HLA, human leukocyte antigen; Tx, transplant.

Donor Factors	MP (n=78)	CS (n=101)	P
Age (yrs)	62.2±6.9	60.6±6.1	0.0905
H/O hypertension	71.8%	74.3%	0.7123
Donor death: CVS cause	81.6% (n = 76)	84.2%	0.6507
Terminal creatinine (mg/dl)	1.3±0.6 (n = 71)	1.2±0.4 (n = 93)	0.0108
CIT (hours)	28.9±10.0	24.0±7.9	0.0003
Import kidney	69 (n=73)	90 (n=99)	0.56
KDPI	88±8.8 (n=77)	87±8.2 (n=90)	0.35‡‡
Recipient Factors			
Age (yrs)	60.7±9.9	62.2±9.1	0.2817
Sex (M/F)	46/32 (59.0%/41.0%)	61/40 (60.4%/39.6%)	0.8475
Race (white/nonwhite)	63/15 (80.8%/19.2%)	76/25 (75.3%/24.8%)	0.3792
ELISA anti-HLA Class I	3.0 (0.0, 7.0)	3.0 (0.0, 6.0) (n = 73)	0.8034**
ELISA anti-HLA Class II	2.0 (0.0, 6.0)	1.0 (0.0, 4.0) (n = 73)	0.2118**
First Tx / Re-Tx	68/10 (87.2%/12.8%)	93/8 (92.1%/7.9%)	0.2798
HLA mismatches	4.0 (4.0,5.0) (n = 72)	5.0 (4.0, 6.0) (n = 91)	0.0629**
Zero antigen mismatches	3	3	1.0
Dual kidney transplants	21	16	0.07
Not on Dialysis	16	13	0.18
Time on Dialysis [years; median (Q1, Q3)]	1.7 (0.7,3.9)	1.6 (0.8,2.9)	0.51‡‡

**Table 2:** Causes of renal failure MP, machine perfused; CS, cold storage; FSGS, focal segmental glomerulosclerosis; GN, glomerulonephritis; CNI, calcineurin inhibitor; NS, not significant.

Disease	MP (n=78)	CS (n=101)	P
Hypertension	18	33	0.16
Diabetes mellitus	22	26	0.58
Polycystic disease	11	14	0.84
Other	5	6	NS
Undetermined	4	4	NS
FSGS	4	4	NS
Autoimmune	4	3	NS
Chronic GN/Membrane GN	2	1	NS
Focal proliferative GN	0	1	NS
Sarcoidosis	1	1	NS
Amyloidosis	0	1	NS
Interstitial nephritis	0	1	NS
IgA nephropathy	1	3	NS
Vesicoureteral reflux	0	2	NS
Obstructive Uropathy	3	0	NS
CNI nephropathy	0	1	NS
Acute tub necrosis	2	0	NS
Pyelonephritis/Nephrolithiasis	1	0	NS
Total	78	101	

Table 3 shows the preservation characteristics of the MP kidneys. Most of the MP kidneys were imported (69 imported, four locally procured, five unknown).

**Table 3:** Preservation characteristics of machine perfused kidneys MP, machine perfused; CS, cold storage

Procurement site	Preservation	n
Local	(CS+MP) at our center	4
Imported	CS elsewhere+ MP at our center	50
	(CS+MP) elsewhere+ MP at our center	14
	MP elsewhere + MP our center	1
	(CS+MP) elsewhere	4
Unknown	CS (elsewhere or at our center) + MP at our center	5

The outcomes are shown in Table 4, Table 5, Table 6, Table 7 and Table 8.

**Table 4:** Outcome parameters **Mann-Whitney, results reported as median (Q1, Q3) +Linear mixed model for repeated measures, results reported as means and 95% CI ‡‡Evaluated using Wilcoxon Rank Sum Test Abbreviations: MP, machine perfused; CS, cold storage; BPAR, biopsy-proven acute rejection; CI, confidence interval

Parameters	MP (n=78)	CS (n=101)	P
Delayed graft function	20.8% (n = 72)	25.8% (n = 97)	0.4550
Length of stay (days)	4.0 (3.0, 6.0) (n = 75)	4.0 (3.0, 6.0)	0.6607**
Readmitted in 3 months	14.1%	11.9%	0.6597
BPAR	38.5%	38.6%	0.98
Time to first BPAR [days; (Q1, Q3)]	236.0 (207.0,418.0)	314.5 (217.0,752.0)	0.009‡‡
Mean length of follow up (years)	4.2±1.9	5±3	0.05
Alemtuzumab induction	78 (100%)	101 (100%)	
Creatinine (mg/dl)	Mean (95% CI)	Mean (95% CI)	0.1995^+^
Creat 14 days	2.9 (2.7, 3.0)	3.0 (2.8, 3.2)	
Creat 3 months	2.4 (2.3, 2.6)	2.5 (2.4, 2.7)	
Creat 6 months	2.1 (2.0, 2.2)	2.2 (2.1, 2.3)	
Creat 3 years	1.7 (1.5, 1.8)	1.7 (1.6, 1.8)	
Creat 4 years	2.0 (1.8, 2.2)	2.1 (1.9, 2.2)	
Creat 5 years	2.0 (1.9, 2.2)	2.1 (2.0, 2.3)	
Creat 6 years	1.5 (1.3, 1.7)	1.5 (1.3, 1.8)	

**Table 5:** Graft and patient survival ++Log-rank test, results reported as estimations from the Kaplan-Meier curve Abbreviations: MP, machine perfused; CS, cold storage

	MP (n=78)	CS (n=101)	p
Graft Survival (non-death censored)			0.2232++
Graft Survival 1 year	87.1%	82.2%	
Graft survival 3 years	71.3%	65.4%	
Graft survival 4 years	66.5 %	59.5 %	
Graft survival 5 years	64.3%	54.3%	
Graft survival 6 years	64.3%	51.5%	
Patient survival			0.1081++
Patient survival 1 year	96.1%	88.1%	
Patient survival 3 years	85.9%	76.7%	
Patient survival 4 years	82.6 %	71.0 %	
Patient survival 5 years	80.6%	67.2%	
Patient survival 6 years	74.0%	63.2%	
Graft Survival (death-censored)			0.39 ^++^
Graft survival 1 year	90.9%	87.8%	
Graft survival 3 years	75.7%	73.8%	
Graft survival 4 years	72.2%	69.7%	
Graft survival 5 years	72.2%	63.6%	
Graft survival 6 years	72.2%	60.3%	

**Table 6:** Causes of graft loss Abbreviations: MP, machine perfused; CS, cold storage

Cause of graft loss	MP	CS
Chronic allograft nephropathy	3	17
Died with functioning graft	11	19
Acute rejection	3	4
Primary non-function	2	3
Technical failure	0	2
Unknown	6	7
Renal disease; new primary	1	0
No graft failure	52	49
Total	78	101

 

**Table 7:** Causes of death Abbreviations: MP, machine perfused; CS, cold storage; ARDS, adult respiratory distress syndrome; CVA, cerebrovascular accident.

Cause of death	MP	CS	
Cardiovascular	6		7
Sepsis; multiple system organ failure	0		7
Liver failure	0		1
ARDS; pneumonia; liver failure	1		5
Cancer	0		2
Suicide	0		1
Encephalopathy	0		1
Pulmonary embolism	1		1
CVA	2		0
Unknown	6		17

**Table 8:** Incidence of viral infections Figures in parentheses represent percentages. MP, machine perfused; CS, cold storage; CMV, cytomegalovirus; EBV, Epstein-Barr virus; NS, not significant.

Virus	MP (n=78)	CS (n=101)	P
CMV	8 (10%)	12 (12%)	NS
Polyoma	19 (24%)	14 (14%)	0.13
EBV	5 (6.4%)	10 (10%)	NS

**Figure 1:**
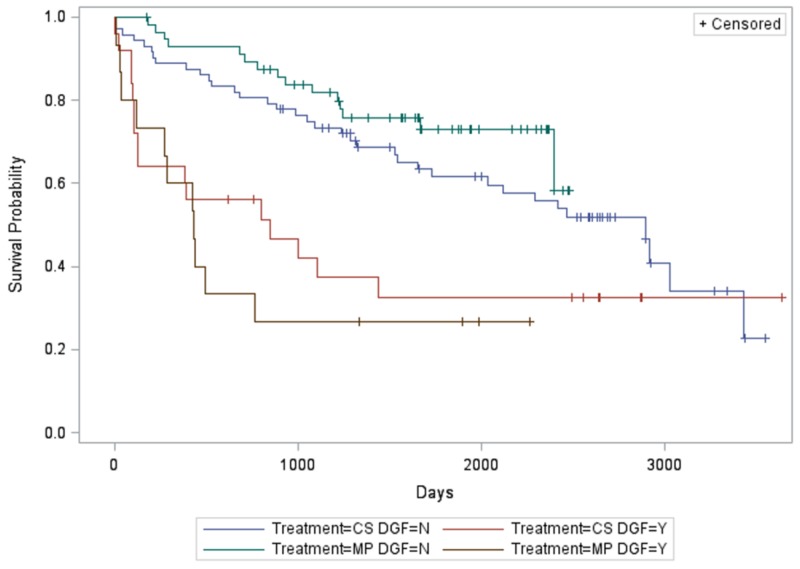
Relationship between treatment arm, delayed graft function, and graft (non-death-censored) survival

There was a significant association between DGF, treatment arm, and graft (non-death-censored) survival (P < 0.0001). Specifically, MP subjects without DGF had longer graft survival compared to CS subjects with DGF (P < 0.0032) and MP subjects with DGF (P < 0.0005) (Figure 1). On adjusted analysis, MP subjects had longer graft survival than CS subjects when DGF was not present.

**Figure 2:**
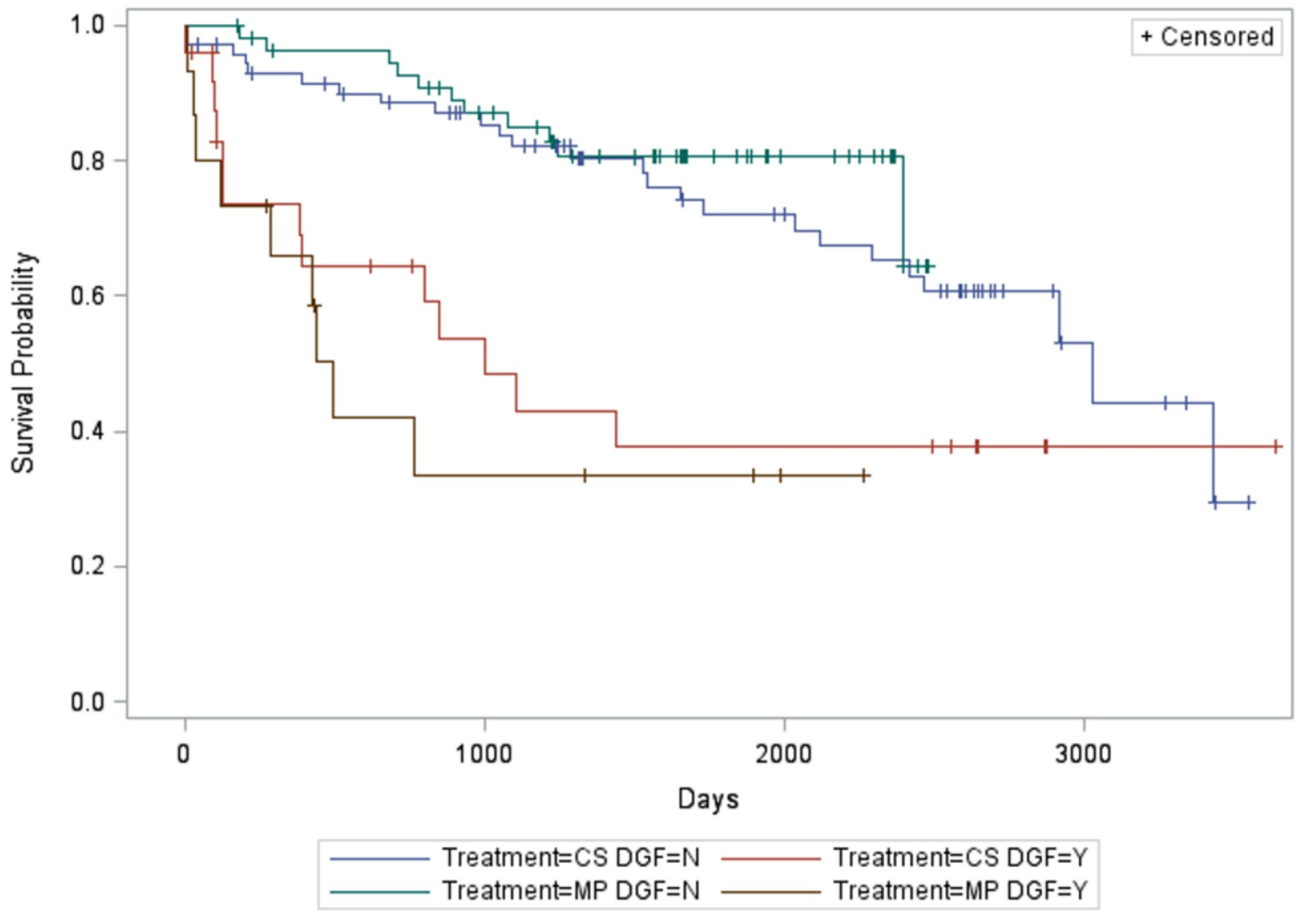
Relationship between treatment arm, delayed graft function, and graft (death-censored) survival

There was a significant association between DGF, treatment arm, and graft (death-censored) survival (P < 0.0001). Specifically, MP subjects without DGF had longer death-censored graft survival compared to CS subjects with DGF (P < 0.0077) and MP subjects with DGF (P < 0.0016) (Figure 2).

**Figure 3:**
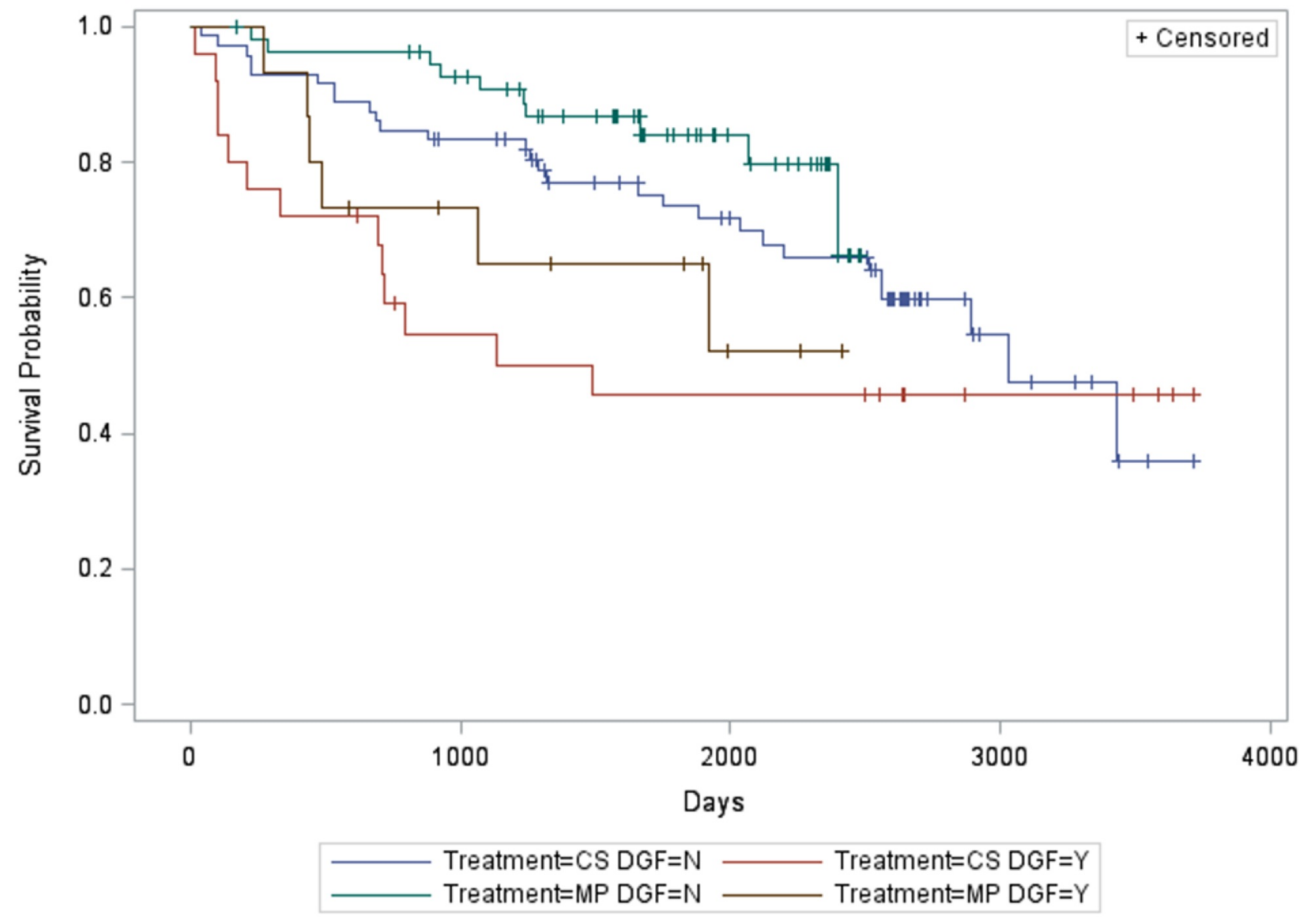
Relationship between treatment arm, delayed graft function, and patient survival

There was a significant association between DGF, treatment arm, and patient survival (P < 0.0360). Specifically, MP subjects without DGF had longer patient survival compared to CS subjects with DGF (P < 0.0289). There were no other significant differences between groups (Figure 3).

DGF

Time on dialysis was significantly associated with DGF (P < 0.0052). Specifically, with each 30-day increase in time on dialysis, the odds of DGF increased by 2% (odds ratio [OR], 1.02; 95% confidence interval [CI]: 1.01, 1.04). There was no difference in the time spent on dialysis between the MP and CS subjects (Table 1). There was no significant association between kidney pumping and DGF (P < 0.1364 MP vs. CS OR, 0.53; 95% CI, 0.23, 1.22).

Graft (non-death-censored) survival

There was a significant interaction between treatment arm and DGF (P < 0.0299). Specifically, MP subjects had reduced risk of graft failure as compared to CS subjects when there was no DGF (hazard ratio [HR], 0.34; 95% CI, 0.17, 0.68). However, there was no significant difference between MP and CS subjects with respect to graft failure when DGF was present (HR, 1.10; 95% CI, 0.46, 2.63). CIT (P < 0.0444) and patient age (P < 0.0176) were also both significantly associated with graft failure. With each one-hour increase in CIT, the risk of graft failure increased by 3% (HR, 1.03; 95% CI, 1.00, 1.06). With each 10-year increase in patient age, the risk of graft failure increased by 42% (HR, 1.42; 95% CI, 1.06, 1.89).

Death-censored graft survival

There was a significant interaction between treatment arm and DGF (P < 0.0497). Specifically, MP subjects had reduced risk of death-censored graft failure as compared to CS subjects when there was no DGF (HR, 0.44; 95% CI, 0.19, 1.00). However, there was no significant difference between MP and CS subjects with respect to death-censored graft failure when DGF was present (HR, 1.49; 95% CI, 0.60, 3.73).

Creatinine

There was a significant three-way interaction between treatment arm, urologic complications (such as urinary leak, ureteric stricture, vesico-ureteral reflux), and acute rejections (P < 0.0014). There was an initial decrease in creatinine followed by an increase in creatinine later in time. The main effects of donor history of diabetes (P < 0.0372), donor history of hypertension (P < 0.0076), recipient diabetes (P < 0.0198), recipient hypertension (P < 0.0057), and vascular complications (P < 0.0412) were significantly associated with creatinine.

Recipients of kidneys from donors with a history of diabetes, donors without a history of hypertension, recipients with diabetes, recipients without hypertension, and recipients with vascular complications all had significantly higher levels of creatinine.

## Discussion

This study compares outcomes of ECD kidney transplantation in a subset of MP kidneys versus CS kidneys. All the kidneys in the MP and CS groups were transplanted under the same immunosuppressive protocol by the same group of surgeons and were cared for by the same set of transplant physicians. Recipient evaluation was done using uniform criteria, and so were the methods used to evaluate the kidneys prior to transplant. Finally, we have presented outcomes after a long duration of follow-up (i.e., six years).

Because the rates of DGF, graft (non-death-censored) and patient survival, and serum creatinine are similar at six years in the MP and CS groups (Tables 4-5), in spite of longer CIT, higher donor creatinine levels and similar KDPI in the MP group, our results support sharing DD kidneys among organ procurement organizations some distance apart, if a portable kidney perfusion machine can be used during transport.

The CITs are long in both the CS and MP groups. This is because of the large proportion of imported kidneys that were transplanted; 69 of 73 (five unknown) in the MP group, and 90 of 99 (two unknown) in the CS group. Once the kidneys arrived at our center, they were subjected to biopsy in both groups, cytotoxic crossmatch was done, and machine perfusion in the MP group, further adding to CITs. Most of the kidneys in the MP group had received a combination of MP and CS (77 of 78), with only one kidney having undergone MP only. Seventy-four of 78 kidneys in the MP group were machine perfused just at our facility immediately prior to transplantation (Table 3). This is referred to as end-ischemic hypothermic machine perfusion (eHMP) [18]. In a paired kidney analysis of eHMP versus CS, eHMP was an important factor for the prevention of DGF. Hence, eHMP is a promising reconditioning technique to improve the quality and acceptance rate of suboptimal grafts.

In a meta-analysis of randomized trials of MP versus CS of donation after brain death donors [19], there was no significant difference in the rates of DGF between the two groups (although the randomized trial in Europe did show a decrease in DGF in the machine perfused kidneys). In a meta-analysis of non-randomized trials, two studies found a significant reduction in DGF following MP versus CS, while the remaining nine did not [19]. In a retrospective review of UNOS data, in the overall cohort, rates of DGF were similar between MP and CS kidneys. However, MP was associated with significantly lower rates of DGF than CS in a propensity-matched cohort (21.1% vs. 29.1%; p < 0.001) as well as on paired kidney analysis (19.7% vs. 27.5%; p<0.001) [20].

Primary non-function (PNF) has been defined as a graft that failed to give one-month dialysis-free survival excluding losses attributable to rejection or vascular thrombosis [21]. We found a similar incidence of PNF in the MP group (2 of 78, 2.5%) compared to the CS group (3 of 101, 3%). There was no significant difference in the incidence of PNF between MP and CS kidneys in a systematic review of three RCTs [21] or in a meta-analysis of seven RCTs [20]. However, the results of the ECD transplants in thec[13] showed PNF was higher in the CS group than in the MP group [p = 0.04].

Several randomized and non-randomized trials have analyzed graft and patient survival statistics of DD kidney transplantation following MP versus CS. Similar graft survival at three years in MP and CS groups has been reported in a UNOS database study [8]. In the Eurotransplant randomized control trial performed on ECD kidneys, similar graft and patient survival were reported at one year [13], but, at three years, better graft survival was reported in the MP group [22].

In an unadjusted analysis, we found no significant differences in graft survival or death-censored graft survival between the MP and CS groups at six years (Table 5). However, the effect of treatment arm on survival changed when DGF was considered. This suggests an interaction between treatment and DGF. Specifically, the MP group without DGF had better graft survival than CS without DGF. This appears to be in concordance with our finding that MP kidney transplants without DGF had reduced risk of graft failure as compared to CS kidney transplants without DGF.

In an unadjusted analysis, we did not find a significant difference in patient survival between MP and CS groups at six years (Table 5). Specifically, the MP group without DGF had better patient survival than the CS group with DGF (Figure 3).

The main purpose of DKT has been to expand the donor pool and to prevent the waste of DD kidneys [11]. MP has helped in transplanting ECD kidneys as DKT by assessing the quality of the kidneys and by helping to “resuscitate” these kidneys from the effects of long CS. We used the Cockcroft-Gault formula to estimate the glomerular filtration rate and the Remuzzi histopathology score prior to doing DKT [23]. This may have led to more DKT and less wastage of a scarce resource in the MP group (21 of 78; 26.9%) than the CS group (16 of 101; 16%; p = 0.07).

One study used SRTR data to identify the role of pulsatile perfusion across different CITs. The adjusted odds of DGF were lower with MP compared with CS for ECD kidneys when CIT was longer than six hours [24]. MP modifies the impact of CITs on DGF but does not eliminate its association with DGF [24]. The optimal strategy to reduce DGF is to minimize CIT and utilize MP in all DD transplants.

A retrospective analysis, a single-center, observational study from the University of Miami found only 19.2% of kidneys were imported, and 10.9% were ECD kidneys [25]. The mean CS time in this study was 6.6 ± 4.5 hours, and mean pump time was 26.7 ± 8.4 hours. The overall DGF rate was 4.4% [25].

In our cohort of MP patients, the mean static CS time was 14 ± 4.1 hours, and mean MP time was 11.2 ± 6.3 hours with a DGF rate of 20.8%. Our cohort of MP kidney transplants had 94.5% (five unknown) import kidneys (thereby longer CIT) and 100% ECD kidneys (which also experience a higher incidence of DGF). In other reports, DGF rates for 114 and 96 ECD MP recipients was 11% [9] and 22% [13], respectively. An important factor that affects the incidence of DGF in these ECD kidneys undergoing eHMP is the length of static CS time in the overall CIT. A longer proportion of static CS time leads to a higher rate of DGF. In a study of paired kidney allografts procured locally, the DGF rate in MP kidneys with short periods of static CS was 5% [26]. It appears that keeping static CS times shorter than five or six hours, even for eHMP preserved kidney transplants, will lead to a reduced rate of DGF. Static CS times can be minimized in the process of sharing ECD kidneys between UNOS donor service areas (DSA) by transporting DD kidneys on MP using a portable machine.

The majority of the ECD kidneys (except the zero antigen mismatches) that were imported from other DSAs were brought in with “full waivers.” These kidneys were discarded by other centers and utilized at our transplant center after having been screened using a biopsy and demonstrating acceptable “pump parameters.”

We used a uniform immunosuppression regimen consisting of alemtuzumab (Campath 1H) induction and tacrolimus monotherapy for all patients in the MP and CS groups. From the very beginning of the use of Campath 1H at our program, we noted a very low incidence of acute rejections, most likely due to its long lymphoid depleting effect [17]. Studies done using alemtuzumab induction and tacrolimus monotherapy have shown lower biopsy-proven acute rejections relative to alemtuzumab and dual/triple immunosuppression (20% of 65 versus 32% of 66, respectively; p = 0.09) [27].

Our study has a few drawbacks, one of which was the lack of randomization. This was a retrospective study of prospectively collected data. However, we did not conduct matched-pair analysis or propensity scoring [20]. We did not use the same type of pump for perfusion throughout the study. We also did not evaluate biomarkers in the perfusate to predict the function of the transplanted kidney [28]. Finally, our study examined the outcomes of transplantation in an era prior to the implementation of kidney allocation using the KDPI.

However, our study was conducted in a “real world” situation of ECD kidney allocation and sharing under the aegis of UNOS. This study was done on ECD kidneys mostly discarded by many centers and eventually transplanted at a large transplant center. We report a longer follow-up period (i.e., six years) than similar studies. 

This study highlights several important findings from transplanting ECD DD kidneys. ECD kidneys must be utilized for transplantation. Despite poorer outcomes, use of kidneys from donors with co-morbidities and/or advanced age targeted to a specific population may provide better survival than those waiting patients remaining on dialysis [23].

Using such kidneys for transplantation requires the implementation of several strategies [23]. Many DD kidneys that can offer survival benefits are declined by transplant programs in the DSA locally and are discarded. Several smaller programs may hesitate to use such so-called “high-risk” kidneys due to the adverse effect the outcomes may have on the program-specific quality reports (PSR). It is apparent that the use of ECD kidneys and high KDPI (>85) kidneys will tend to be concentrated at larger centers that are experienced in the care of elderly recipients of such transplants and where a few adverse outcomes from doing such transplants may not affect the PSR. To ensure efficient and timely use of such DD kidneys, sharing across geographic boundaries must be expedited so that such organs can be efficiently allocated to programs that can use them successfully [29].

Most of the MP kidneys in our study were imported (69 of 73, five unknown) and most of the MP kidneys had been subjected to static CS (77 of 78) prior to eHMP. The mean static CS time was 14 ±4.1 hours, and the rate of DGF was 20.8%. In another study where only 19.2% of kidneys were imported and the mean static CS time 6.6 ± 4.5 hours, the rate of DGF was only 4.4% [25]. Although only 10.9% of kidneys transplanted in that study were ECDs [25], it still is possible that the duration of static CS adversely influences the rate of DGF.

An RCT within UNOS comparing ECD kidneys transported on an MP pump versus transport using static CS would help determine the best method of transportation of ECD kidneys.

## Conclusions

We conducted a study of ECD DD kidney transplants done at a major transplant center, using kidneys mostly “imported” from outside our DSA. We found no difference in the rates of DGF, creatinine levels, as well as six-year graft and patient survival in the cold-stored kidneys versus the eHMP kidneys, in spite of higher risk characteristics of the eHMP cohort. In addition, the recipients of eHMP kidneys had longer graft survival than CS subjects when DGF was not present. The risks of graft failure and death-censored graft failure were significantly lower in the MP ECD kidneys without DGF versus cold stored DD kidneys without DGF. Efforts must be made to reduce the rates of DGF in the “imported” ECD kidneys. One method of doing so may be to machine perfuse the kidney during transportation. It is important to prove the advantages, if any, of such a strategy, by conducting additional RCTs.

## Appendices

Amit Basu made substantial contributions to the conception and design, analysis and interpretation of data, as well as drafting of the article.

Lisa M. Rosen made substantial contributions to analysis and interpretation of data and drafting of the article.

Henkie P. Tan made substantial contributions to conception and design, revising article critically for important intellectual content.

Joanna Fishbein made substantial contributions to the drafting of the article as well as analysis and interpretation of data.

Christine M. Wu made substantial contributions to analysis and interpretation of data, revising article critically for important intellectual content.

Joseph B. Donaldson made substantial contributions to the acquisition of data, analysis, and interpretation of data.

Susan Stuart made substantial contributions to the acquisition of data, revising article critically for important intellectual content.

Nirav A. Shah made substantial contributions to revising article critically for important intellectual content.

Jerry McCauley made substantial contributions to revising article critically for important intellectual content.

Abhinav Humar made substantial contributions to conception and design, revising article critically for important intellectual content.

Ron Shapiro made substantial contributions to conception and design, revising article critically for important intellectual content.

All authors read and approved the final manuscript.

## References

[1] Metzger RA, Delmonico FL, Feng S, Port FK, Wynn JJ, Merion RM (2003). Expanded criteria donors for kidney transplantation. Am J Transplant.

[2] Lloveras J, Arcos E, Comas J, Crespo M, Pascual J (2015). A paired survival analysis comparing hemodialysis and kidney transplantation from deceased elderly donors older than 65 years. Transplantation.

[3] Hart A, Smith JM, Skeans MA (2018). OPTN/SRTR 2016 annual data report: kidney. Am J Transplant.

[4] Schnitzler MA, Whiting JF, Brennan DC (2003). The expanded criteria donor dilemma in cadaveric kidney transplantation.. Transplantation.

[5] Merion RM, Ashley VB, Wolfe RA (2005). Deceased-donor characteristics and the survival benefits of kidney transplantation. JAMA.

[6] Delmonico FL, Burdich JF (2006). Maximizing the success of transplantation with kidneys from older donors. N Eng J Med.

[7] Sung RS, Christensen LL, Leichtman AB (2008). Determinants of discard of expanded criteria donor kidneys: Impact of biopsy and machine perfusion. Am J Transplant.

[8] Matsuoka L, Shah T, Aswad S (2006). Pulsatile perfusion reduces the incidence of delayed graft function in expanded criteria donor kidney transplantation. Am J Transplant.

[9] Stratta RJ, Moore PS, Farney AC (2007). Influence of pulsatile preservation on outcomes of kidney transplantation from expanded criteria donors. J Am Coll Surg.

[10] Stratta RJ, Rohr MS, Sundberg AK (2006). Intermediate-term outcomes with expanded criteria deceased donor kidneys in transplantation. A spectrum or specter of quality?. Ann Surg.

[11] Moore PS, Farney AC, Sundberg AK (2007). Dual kidney transplantation: A case control comparison with single kidney transplantation from standard and expanded criteria donors. Transplantation.

[12] Moers C, Smits JM, Maathius MHJ (2009). Machine perfusion or cold storage in deceased donor kidney transplantation. N Eng J Med.

[13] Treckmann J, Moers C, Smits JM (2011). Machine perfusion versus cold storage for preservation of kidneys from expanded criteria donors after brain death. Transplant Int.

[14] KDPI calculator. (2018) (2019). Organ Procurement and Transplantation Network. KDPI calculator. (2018). https://optn.transplant.hrsa.gov/resources/allocation-calculators/kdpi-calculator/.

[15] Remuzzi GR, Cravedi P, Perna A (2006). Long term outcomes of renal transplantation from older donors. N Engl J Med.

[16] Escofet X, Osman H, Griffiths DFR, Woydag S, Jureqicz AW (2003). The presence of glomerular sclerosis has a significant effect on function after cadaveric renal transplantation. Transplantation.

[17] Shapiro R, Basu A, Tan HP (2005). Kidney transplantation under minimal immunosuppression after pre-transplant lymphoid depletion with Thymoglobulin or Campath. J Am Coll Surg.

[18] Gallinat A, Amrilaeva V, Hoyer DP (2017). Reconditioning by end-ischemic hypothermic machine perfusion. Clin Transplant.

[19] O’Callaghan JM, Morgan RD, Knight SR, Morris PJ (2013). Systemic review and meta-analysis of hypothermic machine perfusion versus static cold storage of kidney allografts on transplant outcomes. Br J Surg.

[20] Cannon RM, Brock GN, Garrison RN, Smith JW, Marvin MR, Franklin GA (2013). To pump or not to pump: A comparison of machine perfusion versus cold storage for deceased donor kidney transplantation. J Am Coll Surg.

[21] Lam VW, Laurence JM, Richardson AJ, Pleass HCC, Allen RD (2013). Hypothermic machine perfusion in deceased donor kidney transplantation. J Surg Res.

[22] Gallinat A, Moers C, Smits JM (2013). Machine perfusion versus static cold storage in expanded criteria donor kidney transplantation: 3 year follow up data. Transplant Int.

[23] Péréz-Sáez MJ, Montero N, Redondo-Páchon D, Crespo M, Pascual J: Strategies for an expanded use of kidneys from elderly donors (2017). Transplantation.

[24] Gill J, Dong J, Eng M, Landsberg D, Gill JS (2014). Pulsatile perfusion reduces the risk of delayed graft function in deceased donor kidney transplants irrespective of donor type and cold ischemia times. Transplantation.

[25] Ciancio G, Gaynor JJ, Sageshima J (2010). Favorable outcomes with machine perfusion and long pump times in kidney transplantation: A single center observational study. Transplantation.

[26] Shah AP, Milgrom DP, Mangus RS, Powelson JA, Goggins JC, Milgrom ML (2008). Comparison of pulsatile perfusion and cold storage for paired kidney allografts. Transplantation.

[27] Margreiter R, Klempnauer J, Neuhaus P, Muehlbacher F, Boesmueller C, Calne RY (2008). Alemtuzumab (Campath-1H) and tacrolimus monotherapy after renal transplantation: results of a prospective, randomized trial. Am J Transplant.

[28] Parikh CR, Hall IE, Bhangoo RS (2016). Association of perfusate biomarkers and pump parameters with delayed graft function and deceased donor kidney allograft function. Am J Transplant.

[29] Axelrod DA, Friedewald JJ (2016). Utilizing high-risk kidneys:risks, benefits and unintended consequences. Am J Transplant.

